# Occurrence and Genomic Characterization of *mcr-1*-Harboring *Escherichia coli* Isolates from Chicken and Pig Farms in Lima, Peru

**DOI:** 10.3390/antibiotics11121781

**Published:** 2022-12-08

**Authors:** Dennis Carhuaricra, Carla G. Duran Gonzales, Carmen L. Rodríguez Cueva, Yennifer Ignacion León, Thalia Silvestre Espejo, Geraldine Marcelo Monge, Raúl H. Rosadio Alcántara, Nilton Lincopan, Luis Luna Espinoza, Lenin Maturrano Hernández

**Affiliations:** 1 Research Group in Biotechnology Applied to Animal Health, Production and Conservation [SANIGEN], Laboratory of Biology and Molecular Genetics, Faculty of Veterinary Medicine, Universidad Nacional Mayor de San Marcos, Lima 15021, Peru; 2 Programa de Pós-Graduação Interunidades em Bioinformática, Instituto de Matemática e Estatística, Universidade de São Paulo, Rua do Matão 1010, São Paulo 05508-090, Brazil; 3 Department of Clinical Analysis, School of Pharmacy, University of São Paulo, São Paulo 05508-000, Brazil; 4 Department of Microbiology, Institute of Biomedical Sciences, University of São Paulo, São Paulo 05508-000, Brazil

**Keywords:** *mcr-1* gene, colistin, chicken farm, pig farm, *Escherichia coli*

## Abstract

Resistance to colistin generated by the *mcr-1* gene in *Enterobacteriaceae* is of great concern due to its efficient worldwide spread. Despite the fact that the Lima region has a third of the Peruvian population and more than half of the national pig and poultry production, there are no reports of the occurrence of the *mcr-1* gene in *Escherichia coli* isolated from livestock. In the present work, we studied the occurrence of *E. coli* carrying the *mcr-1* gene in chicken and pig farms in Lima between 2019 and 2020 and described the genomic context of the *mcr-1* gene. We collected fecal samples from 15 farms in 4 provinces of Lima including the capital Lima Metropolitana and recovered 341 *E. coli* isolates. We found that 21.3% (42/197) and 12.5% (18/144) of the chicken and pig strains were *mcr-1*-positive by PCR, respectively. The whole genome sequencing of 14 *mcr-1*-positive isolates revealed diverse sequence types (e.g., ST48 and ST602) and the presence of other 38 genes that confer resistance to 10 different classes of antibiotics, including beta-lactamase *bla*_CTX-M-55_. The *mcr-1* gene was located on diverse plasmids belonging to the IncI2 and IncHI1A:IncHI1B replicon types. A comparative analysis of the plasmids showed that they contained the *mcr-1* gene within varied structures (*mikB*–*mcr1*–*pap2,* IS*Apl1*–*mcr1*–*pap2,* and Tn6330). To the best of our knowledge, this is the first attempt to study the prevalence of the *mcr-1* gene in livestock in Peru, revealing its high occurrence in pig and chicken farms. The genetic diversity of *mcr-1*-positive strains suggests a complex local epidemiology calling for a coordinated surveillance under the One-Health approach that includes animals, retail meat, farmers, hospitals and the environment to effectively detect and limit the spread of colistin-resistant bacteria.

## 1. Introduction

Antimicrobial resistance (AMR) represents a growing threat to global health, principally in developing countries, where the high population density, poor medical care and unregulated use of antibiotics provide a favorable environment for the emergence and dissemination of multidrug-resistant bacteria (MDR) [1]. The increased prevalence of bacterial pathogens resistant to last-line antibiotics (carbapenems, colistin and tigecycline) raises serious concerns about our ability to treat infectious diseases in humans and animals [2]. Colistin is one of the last-resort treatments against multidrug-resistant strains of *Enterobacterales*, but its unregulated overuse as a therapeutic drug and growth promoter in pig and poultry farming has favored the emergence of colistin-resistant strains [3,4]. Since the discovery of the plasmid-encoded colistin resistance gene named *mcr-1* in China in 2015 [5], this gene has been described in human, animal and environmental samples around the world [6]. The rapid spread of the *mcr-1* gene by efficient horizontal transfer is driven by the IncI2, IncHI2 and IncX4 plasmids [4].

*Escherichia coli* is a commensal bacterium that inhabits the gastrointestinal tract of humans and animals and represents a major reservoir of antimicrobial resistance genes (ARGs), mostly acquired through horizontal gene transfer [7,8]. As a result of this capacity, *E. coli* has been commonly used as an indicator to monitor AMR in livestock, food and humans [9,10]. Even though they are normally commensal, certain strains of *E. coli* are associated with infections. For example, pathogenic *E. coli* may cause neonatal and postweaning diarrhea and edema in swine, while it may cause infections of the respiratory tract and soft tissues, resulting in colibacillosis, air sacculitis and cellulitis in chickens [11]. *E. coli* and *Klebsiella pneumoniae* carrying the *mcr-1* gene have recently been reported in isolates from Peruvian hospitals [12,13,14,15], as well as in isolates from slaughtered chickens destined for human consumption [16]. Due to the recurrent detection of resistant enterobacteria in hospitals, the Peruvian government decreed in late 2019 the prohibition of the manufacture, sale and import of veterinary products containing the active compound of colistin (Polymyxin E).

The Lima region has a third of the Peruvian population and concentrates the largest animal production in the country. In fact, by 2020, 53% and 43% of the national poultry and pig production were concentrated in Lima, mainly in farms located on the outskirts of the capital Lima Metropolitana. Reporting the growing colistin resistance and *mcr-1* prevalence in poultry and pig farms in low- and middle-income countries (LMIC) could be of high importance, but until now has been neglected in Peru [17]. Indeed, there is no information about the occurrence of *mcr* genes in livestock in Peru due to the absence of a systematic surveillance. In the present work, we investigated the occurrence of *E. coli* carrying the *mcr-1* gene isolated from chicken and pig farms in Lima, Peru, from 2019 to 2020 and performed a genomic analysis of the isolates carrying *mcr-1* to determine the genetic diversity and phylogenetic relationships of these isolates. Additionally, we characterized the virulence and ARGs profiles and explored the genomic context of the *mcr*-1 gene.

## 2. Methods

### 2.1. Sample Collection and Bacterial Culture

We collected 348 fecal samples from 8 chicken farms and 300 samples from 7 pig farms in 4 provinces of Lima, including the Peruvian capital, Lima Metropolitana, between 2019 and 2020 (Supplementary Table S1). All samples were later organized in pools. One pool was prepared for each chicken shed or pigpen combining 2 g of feces in a 50 mL tube; at least 5 pools per farm were obtained. Each fecal pool was diluted in buffered saline solution (0.9%) and thereafter plated onto MacConkey agar (BD Difco) and incubated overnight at 37 °C. At least five suspected colonies of *E. coli* (lactose-fermenting colonies, convex morphology and pinkish color appearance) were selected from each pool, inoculated on eosin methylene blue agar (EMBA) and incubated for 24 h at 37 °C, observing the growth of typical metallic green colonies. Finally, a 7-parameter biochemical test including Simmon’s citrate agar, lysine iron agar, triple sugar iron, motility–indole–lysine medium, sulfur indole motility medium, urea medium, red methyl and Voges Proskauer medium were used to confirm *E. coli*.

### 2.2. mcr-1 Gene Screening by PCR

All isolates were screened for the *mcr-1* gene using the procedures described by Rebelo et al. (2018) [18]. Shortly, we used forward 5′-AGTCCGTTTGTTCTTGTGGC-3′ and reverse 5′-AGATCCTTGGTCTCGGCTTG-3′ primers with 2 µL of 10X PCR Buffer (100 mM KCl, 100 mM Tris-HCl, 20 mM MgCl_2_), 1.6 µL of deoxynucleotide triphosphate (dNTPs) 10 mM, 0.2 µL of DreamTaq 5 U/µL (Thermo Fisher Scientific, Waltham, MA, USA) and 2 µL of DNA in a final volume of 20 µL. The following condition were used: 1 cycle of initial denaturation at 94 °C for 15 min, followed by 25 cycles of denaturation at 94 °C for 30 s, annealing at 58 °C for 90 s and elongation at 72 °C for 60 s, with a final extension step of 72 °C for 10 min. We used the *mcr-1*-harboring *E. coli* CDC-AR-0346A reference strain (https://www.microbiologics.com/01259P) (accessed on 27 November 2022) as a positive control for all PCR runs.

### 2.3. Whole-Genome Sequencing and Assembly

From all *mcr-1*-positive *E. coli*, we selected 14 isolates for whole-genome sequencing (accession numbers and sequencing statistics are provided in Supplementary Table S2). The DNA was extracted from pure colonies using the PureLink™ Genomic DNA Kit (Invitrogen’, Cat. No K1820-02). DNA concentration was measured using the Qubit dsDNA HS assay (Invitrogen, Cat. No Q33230). Then 1 ng of DNA was used for Nextera XT library preparation and subsequent sequencing using 2 × 250 bp reads on the Illumina Miseq platform (Illumina, San Diego, CA, USA). The quality of the fastq files was evaluated with FastQC v0.11.9 [19], and the trimming of low-quality reads was performed with Trimmomatic v0.39 [20]. Finally, the assembly was performed with SPAdes v3.14.1 [21], and Prokka v1.14.6 [22] was used for genome annotation.

### 2.4. Sequence Analysis

We used the mlst v2.19.0 tool (https://github.com/tseemann/mlst) (accessed on 16 August 2022) and EzClermont v0.6.3 [23] to determine the multilocus sequence type (MLST) and phylogroup type, respectively. ARGs, virulence genes and plasmid replicon types were annotated using the Resfinder, VirulenceFinder and PlasmidFinder databases from the Center for Genomic Epidemiology with the ABRICATE v. 1.0.1 tool (https://github.com/tseemann/abricate) (accessed on 16 August 2022) using the following settings: a nucleotide identity of 80% and minimum coverage of 80%.

For the phylogenetic reconstruction, we used the following pipeline: Snippy v 4.6.0 (https://github.com/tseemann/snippy) (accessed on 15 July 2022) to generate the core-genome alignment of 14 *E. coli* genomes including the reference genome sequence *E. coli* K-12 (Genbank accession: NC_000913.3); given that recombination is widespread in bacteria genomes, Gubbins v3.2 [24] was used to detect and mask recombinant regions, and IQ-TREE v2.0 [25] to construct a maximum-likelihood tree based on a general time-reversible (GTR) nucleotide substitution model with 1000 bootstrap replicates. Tree visualization and annotation were created using the ggtree v3.0.4 [26] package in R 4.2. The genetic context of the *mcr-1*-encoding plasmid sequences was represented using Easyfig v2.2.2 [27].

We used a bioinformatics approach to identify the plasmid sequences. First, we used plasmidSPAdes [28] for plasmid assembly from raw data. Second, after checking if these sequences contained the *mcr-1* cassette, we used plasmidfinder [29] to check if the predicted plasmid has a replicon and then we used oriTfinder [30] to identify the origin of the transfer site (oriT) and conjugative elements. Finally, we performed a search in the PLSDB database [31] to identify similar plasmid sequences. Highly similar sequences were compared with the predicted plasmids to generate a circular view using Blast Ring Image generation (BRIG) software [32].

## 3. Results

### 3.1. Prevalence of the mcr-1 Gene in Poultry and Pig Farms

**Figure 1:**
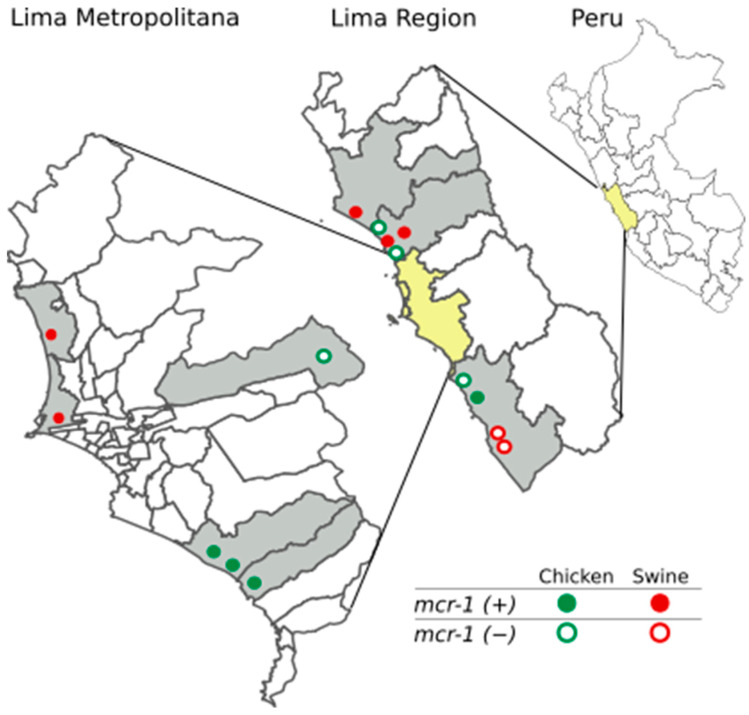
Geographical distribution of pig and poultry farms sampled in Lima, Peru. The location of the farms with the presence of *mcr-1*-positive *E. coli* isolates from chicken and swine is represented by circles filled in green and red, respectively. The location of the farms without the presence of *mcr-1*-positive *E. coli* isolates from chicken and swine is represented by green and red circles, respectively.

A total of 15 farms located in Lima were investigated in this study. We collected 648 fecal samples and recovered 197 *E. coli* isolates from 8 chicken farms and 144 *E. coli* isolates from 7 pig farms. The *mcr-1* gene was identified in four of eight chicken farms in three Lima provinces, and in five of seven pig farms located in three Lima provinces (Figure 1). The occurrence of the *mcr-1* gene was variable: 81% of the isolates were *mcr-1*-positive in the AV8 farm (26/32 isolates), while 52.2% (12/23) were positive in the AV4 farm, and just 10.7% (3/28) were positive in the AV5 farm (see Supplementary Table S1). Overall, the *mcr-1*-specific PCR identified the gene in 21.3% (42/197) of the isolates from poultry and in 12.5% (18/144) of the isolates from pigs.

### 3.2. Genetic Characterization of E. coli Harboring mcr-1 and Resistome

**Figure 2:**
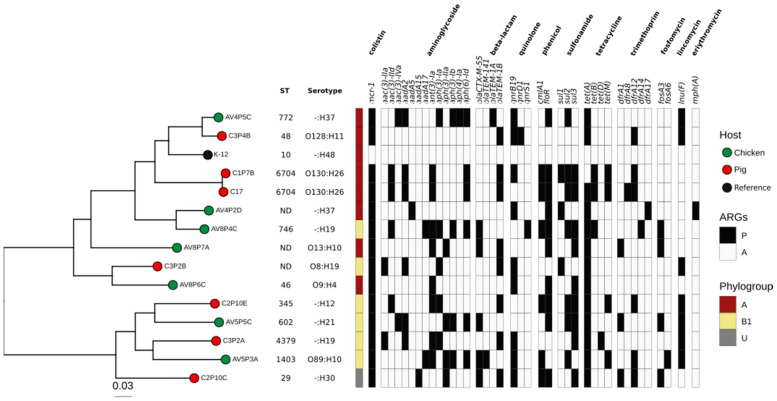
Resistome of 14 *mcr-1*-harboring *E. coli* isolated from pig and poultry farms in Lima, Peru. From left to right: phylogenomic tree based on SNPs of 14 *E. coli* genomes from chicken (circle filled with green) and pig (circle filled with red) farms. The *E. coli* K-12 strain was used as the reference. First column indicates the phylogroups A (red), B (yellow) or U (gray). The heatmap shows the presence/absence (P/A) of ARGs detected in *mcr-1*-positive *E. coli* genomes.

The whole genome of 14 *mcr-1*-positive *E. coli* was sequenced (7 genomes from pigs and 7 from chicken). The sequence size varied from 4.75 to 5.90 Mb. A total of 10 different MLSTs were identified in 11 isolates, while 3 were not determined (Supplementary Table S2). Most isolates were identified as phylogenetic groups A (*n* = 7) and B1 (*n* = 6), while one isolate was typed as an unknown phylogroup (U). There was no differential clustering between the isolates from poultry and porcine sources (Figure 2).

Interestingly, the genome sequence analysis showed a high number of resistance genes. We detected 39 different genes that confer resistance to 10 different classes of antibiotics, including *mcr-1* (Table 1 and Figure 2). Five isolates contained the *bla*_CTX-M-55_ gene for resistance to extended-spectrum beta-lactamase (ESBL). At least 70% of the isolates contained a gene for resistance to ampicillin, chloramphenicol, kanamycin and trimethoprim, and at least 90% of the isolates contained a gene for resistance to streptomycin, sulfisoxazole and tetracycline.

Forty-two genes encoding virulence factors were detected in all *E. coli* genomes using the VirulenceFinder tool v2.0 (see Supplementary Figure S1 and Table S3), including genes related to evasion/invasion (*capU*, *kpsE*, *kpsMII_K5*, *gad*, *iss*, *ompT*, *sepA*, *traT*), toxins (*astA*, *cea*, *cib*, *hlyF*, *stb*, *toxB*), secretion system (*cif*, *espABFJ*, *nleABC*, *terC*, *ti*r), adherence (*eae*, *lpfA*, *perA*, *tsh*) and iron uptake (*fyuA*, *ireA*, *irp2*, *iucC*, *iutA*, *sitA*). The AV5P5C isolate from chicken was classified as APEC because it presented genes encoding outer membrane protein (*ompT*), hemolysin (*hlyF*), increased serum survival (*iss*), aerobactin siderophore receptor (*iutA*), temperature-sensitive hemagglutinin (*tsh*) and siderophores (*IucC*, *sitA*) [33,34].

Additionally, 33 types of plasmid replicons were identified in the analyzed genomes; the most overrepresented was IncFIB, followed by IncX1, ColRNAI, IncFIC(FII) and IncFII(pHN7A8) (Supplementary Figure S1 and Table S3).

### 3.3. Characterization of the Genetic Context of the mcr-1 Gene

**Figure 3:**
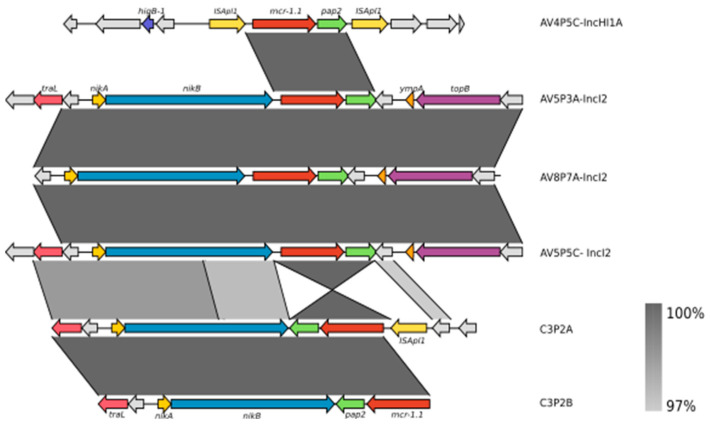
Genetic context of the *mcr-1* gene in *E. coli* genomes isolated from chicken and pig farms in Lima. Six representative sequences show the diversity of the structural context of the *mcr-1* gene from chicken and pig *E. coli* isolates in this study. The *mcr-1* gene is marked in red. IS*Apl1* transposase, *pap2* and *nickB* genes are marked in yellow, green and yellow, respectively. Regions of homology between sequences (>97%) are indicated by the graded shading.

The *mcr-1* sequence of all *E. coli* genomes was 100% identical to the *mcr-1* sequence from the Resfinder database. The exploration of the genetic context of *mcr-1* allowed us to identify three different types of *mcr-1*-containing cassettes suggesting a diverse genetic context of *mcr-1*-harboring *E. coli* in the farms of Lima (Figure 3). Two different context structures were identified in chicken farms. The IS*Apl1–mcr1–pap2–*IS*Apl1* was identified in the AV4P5C isolate, which belongs to ST48. This cassette shows a structure called Tn6330 inserted into an IncHI1A:IncHI1B hybrid plasmid. Tn6330 is a composite transposon that improves the transmission of the *mcr-1* gene [35]. The second structure shows the *nikB–mcr1–pap2* composition that has lost IS*Apl1* both upstream and downstream. This structure was found within IncI2 plasmids in the AV4P2D, AV5P5C, AV5P3A and AV8P7A isolates (Figure 3). Interestingly, a BLASTN search of the AV5P3A plasmid carrying *mcr-1* showed a high similarity of this plasmid to the IncI2 plasmid pkpCOL17 (99% of identity and 99.97% of coverage) identified in *K. pneumoniae* isolated from a patient in a Peruvian hospital [15] (Supplementary Figure S2). In a pig isolate (C3P2A), we observed the presence of a downstream copy of ISApl1 only. Due to short-read sequencing, we were not able to determine the genetic context of the *mcr-1* gene for six isolates because of incomplete assembly.

## 4. Discussion

We studied the occurrence of *E. coli* carrying the *mcr-1* gene in 15 livestock farms in Lima, Peru, from 2019 to 2020. The results of the PCR showed that *E. coli* was positive for *mcr-1* in 9 of the 15 farms evaluated in this study at different rates, i.e., in 21% (42/197) of the isolates from poultry and in 12% (18/144) of the isolates from pigs. To the best of our knowledge, this is the first study to investigate the occurrence of *mcr-1*-positive *E. coli* isolates in farms in Peru. Previous works reported *E. coli* isolates carrying the *mcr-1* gene in samples of clinical and food origin in Peru [12,13,14,16]. In December 2019, the Ministry of Agriculture and Irrigation of Peru (MINAGRI) published a resolution prohibiting the use of colistin in food-producing animals [36]. The effect of the ban was not evaluated in this study because all samples were collected before the application of the resolution in March 2020. We expect a reduction in *mcr-1* prevalence as was observed in China. After the implementation of the colistin prohibition for veterinary use in China, the prevalence of *E. coli* carrying *mcr-1* decreased from 45% to 19% between 2016 and 2018 in pig farms [37]. In South America, the circulation of *mcr-1*-harboring *Enterobacteriaceae* isolates has a higher prevalence in animals (8.7%) than in food (5.4%) or humans (2.0%), mainly in Brazil, Bolivia and Argentina [38].

All isolates sequenced in this study belong to different sequence types, suggesting an important diversity in *mcr-1*-positive *E. coli*. These isolates were classified as belonging to phylogroup A or B1, with commensal *E. coli* usually found in humans and animal hosts [39,40]. The clones found in our study, ST48, ST602, ST746, ST46, ST345, were previously reported in clinical isolates from humans and other hosts; ST602 is widely distributed internationally [41,42]. This information is concerning, since it suggests that these strains have the ability to move and proliferate in different ecological niches, which may facilitate the genetic exchange of the *mcr-1* gene and other antibiotic resistance genes between a wide range of bacterial species. A highly diverse resistome was revealed, with 39 different genes conferring resistance to 15 different antibiotics including ESBL, chloramphenicol, ciprofloxacin, tetracycline and sulfamethoxazole, indicating an extensive circulation of *E. coli* carrying multiple antibiotic-resistant genes in livestock in Lima; in fact, Peru is considered one of the countries with a high projected increase of antimicrobial consumption by livestock [43]. We detected that *mcr-1* was associated with resistance mechanisms to beta-lactams; five *mcr-1*-positive *E. coli* also encoded *bla*_CTX-M-55_, while other eight isolates produced the *bla*_TEM-1B_ gene. The co-occurrence of *mcr-1* and beta-lactam genes was also reported previously in South America, in samples from chicken meat in Brazil and in *E. coli* isolated from pig farms and companion animals in Argentina [44,45,46].

The composite transposon Tn6330 (IS*Apl1*–*mcr1–pap2–*IS*Apl1*) is considered the main vehicle for *mcr-1* mobilization [6,35]. Only one out of fourteen *mcr-1*-positive *E. coli* sequenced in this work contained Tn6330 with both copies of IS*Apl1* within an IncHI1A:IncHI1B plasmid, while in another isolate, we noted the presence of an upstream copy of IS*Apl1*. Cassettes with the Tn6330 structure are generally mobilized by IncHI2 plasmids; however, *E. coli* has been reported harboring *mcr-1* into hybrid plasmids containing the incompatible types IncHI1A and IncHI1B in Asia [47,48]. Up to now, only four plasmids have been described to carry the *mrc-1* gene in Latin America: IncX4, IncP, IncI2 and IncHI2 [49]. On other hand, some *E. coli* genomes from chicken and pigs presented *mcr-1* carried by the IncI2 plasmid lacking the IS*Apl1* copies. The plasmid IncI2 has already been described to spread different *mcr* genes variants in Latin American countries such as Argentina, Brazil and Uruguay [50]. According to global genomic studies, *mcr-1* sequences with two copies of IS*Apl1* are found in lesser frequency than sequences with only a single copy of IS*Apl1*, while the majority of positive *mcr-1* isolates do not present the IS*Apl1* sequence [4,6]. Due to the short-read sequencing method used in this work, we were not able to determine the genetic context of the *mcr-1* gene for six *mcr-1*-positive genomes because of the incomplete assembly of the (plasmid) sequences. Therefore, we cannot exclude the presence of other plasmid types that mobilize the *mcr-1* gene in our isolates.

In conclusion, we determined the occurrence of *mcr-1*-harboring *E. coli* in chicken farms (21.3%) and pig farms (12.5%) in Lima. The genomic analysis showed diverse lineages of *E. coli* carrying the *mcr-1* gene mobilized by the IncI2 and IncHI1A:IncHI1B plasmids, including the presence of IS*Apl1* copies enhancing the dissemination of *mcr-1*. The elevated prevalence of multidrug-resistant strains in farms in Lima could serve as a reservoir of ARGs that can be disseminated by farmers or food, impacting public health. We need to expand the genomic and epidemiological surveillance of colistin resistance in farmers, livestock, the environment, wastewater and hospitals to understand the dynamic of *mcr-1* transmission in Peru.

## Figures and Tables

**Table 1:** ARGs profile of 14 *E. coli* genomes carrying *mcr-1*.

Class	Antibiotics	Number of *mcr-1* + *E. coli* (%)	Gene Name (n)
Aminoglycoside	gentamicin	8 (57)	*aac(3)-IIa (2)*, *aac(3)-IId (4)*, *aac(3)-IVa (1)*, *aac(3)-VIa (1)*
	hygromycin B	1 (7)	*aph(4)-Ia (1)*
	kanamycin	11 (79)	*aph(3’)-Ia (5)*, *aph(3’)-IIa (4)*, *aph(6)-Id (6)*
	streptomycin	13 (93)	*aadA2 (6)*, *aadA5 (1)*, *aadA15 (1)*, *aadA17 (2)*, *ant(3’’)-Ia (8)*, *aph(3’’)-Ib (4)*
Beta-lactam	ampicillin	10 (71)	*blaTEM-1A (1)*, *blaTEM-1B (8)*, *blaTEM-141 (1)*
	ceftriaxone	5 (36)	*blaCTX-M-55 (5)*
Quinolone	ciprofloxacin	10 (71)	*qnrB19 (9)*, *qnrD1 (1)*, *qnrS2 (1)*
Folate pathway antagonist	trimethoprim	10 (71)	*dfrA1 (3)*, *dfrA8 (1)*, *dfrA12 (6)*, *dfrA14 (1)*, *dfrA17 (1)*
Fosfomycin	fosfomycin	5 (36)	*fosA3 (4)*, *fosA6 (1)*
Glycylcycline	tetracycline	14 (100)	*tet(A) (13)*, *tet(B) (3)*, *tet(D) (1)*, *tet(M) (4)*
	lincomycin	7 (50)	*Inu(F) (7)*
Macrolide	erythromycin	1 (7)	*mph(A) (1)*
Phenicol	chloramphenicol	11 (79)	*cmlA1 (6)*, *floR (10)*
Sulphonamide	sulfamethoxazole	13 (93)	*sul1 (3)*, *sul2 (7)*, *sul3 (9)*

## Data Availability

Genome sequence data analyzed in this study can be found here: https://www.ncbi.nlm.nih.gov/bioproject/PRJNA892251.

## Supplementary Materials

The following supporting information can be downloaded at: https://www.mdpi.com/article/10.3390/antibiotics11121781/s1, Figure S1: Virulome and plasmid replicons of 14 *mcr-1 E. coli*.; Figure S2: Genetic characteristics of the Inc2 *mcr-1*-carrying plasmid identified in this study. Circular view and alignment comparison of closely related IncI2 plasmids carrying the *mcr-1* gene.; Supplementary Table S1: Sampling information and *mcr-1* positivity by PCR in the farms sampled in this study.; Supplementary Table S2: Accession numbers and sequencing statistics of 14 *E. coli* genomes carrying the *mcr-1* gene.; Supplementary Table S3: Virulence factors and plasmid types predicted by *abricate* using VFDB (Virulence Factor Database) and Plasmidfinder. Values represent the gene coverage.
