# Supplementary figures and images for: Occurrence and Genomic Characterization of mcr-1-Harboring Escherichia coli Isolates from Chicken and Pig Farms in Lima, Peru
Source: Antibiotics (Basel). 2022 Dec 8;11(12):1781. doi: 10.3390/antibiotics11121781 (PMC9774552; doi:10.3390/antibiotics11121781)

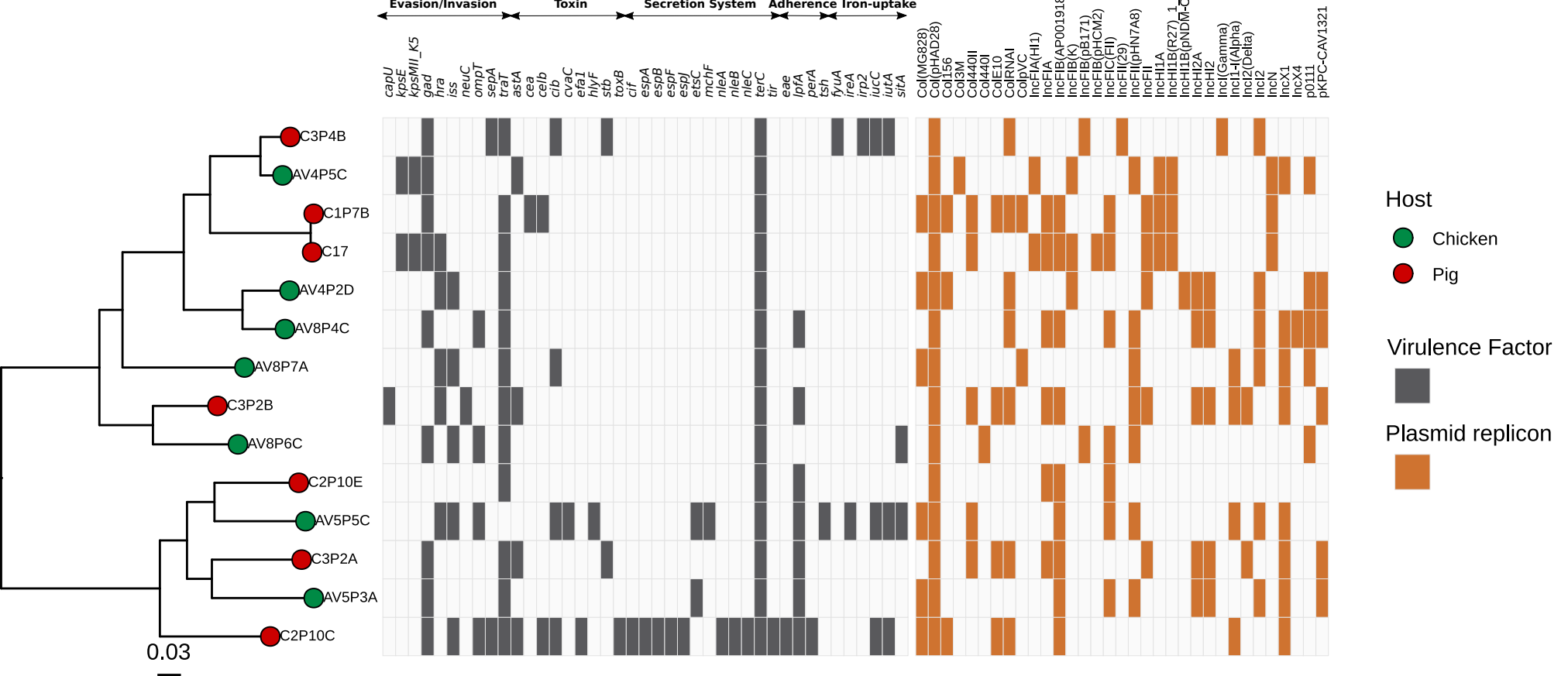

Supplement: Supplementary file 1 [file antibiotics-11-01781-s001.zip › Figure S1.pdf]

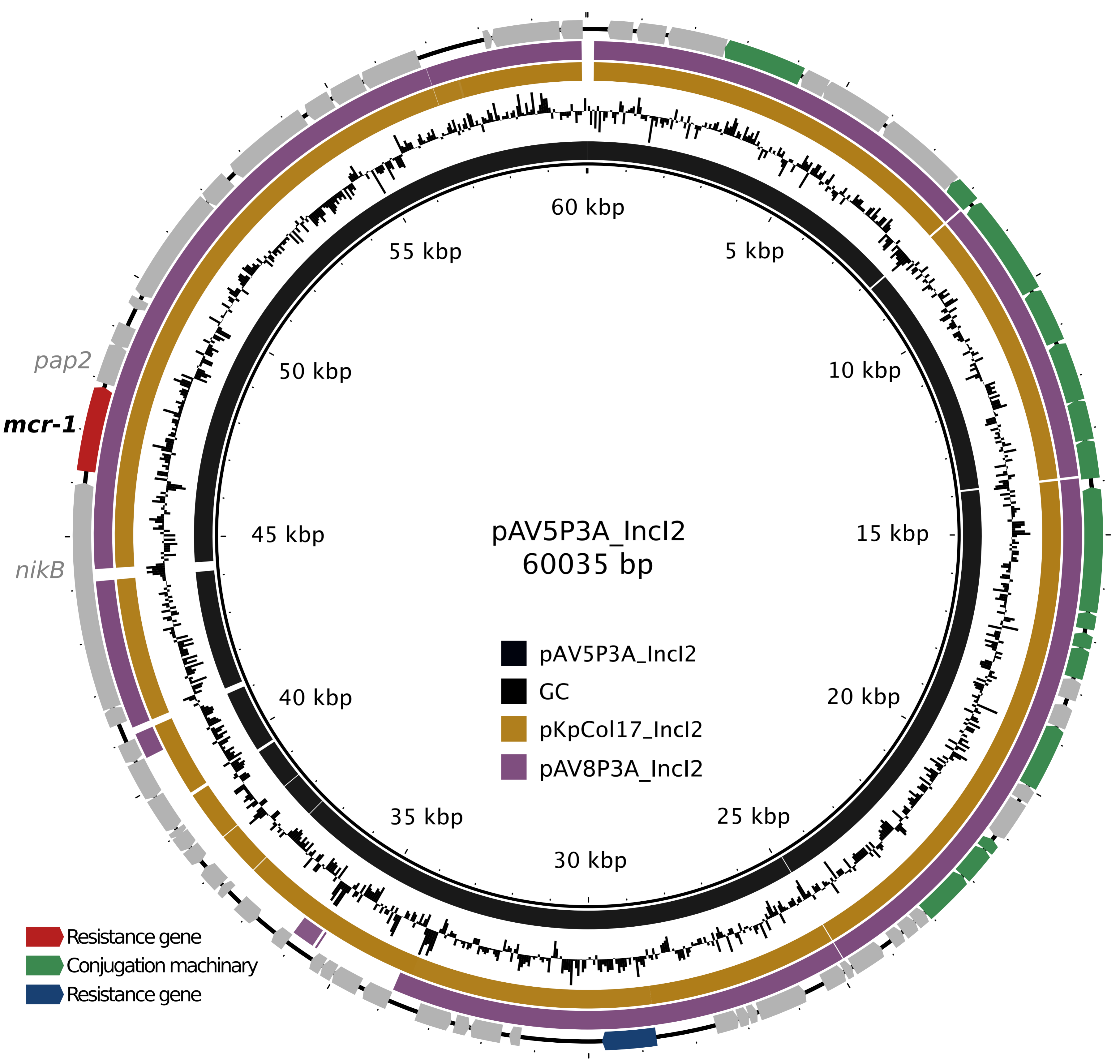

Supplement: Supplementary file 1 [file antibiotics-11-01781-s001.zip › Figure S2.pdf]
